# The utility of SYNTAX score predictability by electrocardiogram parameters in patients with unstable angina

**DOI:** 10.1186/s12872-022-02455-6

**Published:** 2022-01-12

**Authors:** Mohammad Reza Hatamnejad, Amir Arsalan Heydari, Maryam Salimi, Soodeh Jahangiri, Mehdi Bazrafshan, Hamed Bazrafshan

**Affiliations:** 1 Faculty of Medicine, Shiraz University of Medical Sciences, Shiraz, Iran; 2 Shiraz University of Medical Sciences, Shiraz, Iran; 3 Department of Cardiology Medicine, Al-Zahra Charity Hospital, Shiraz University of Medical Sciences, Zand St, PO Box: 71348-14336, Shiraz, Iran

**Keywords:** Unstable angina, SYNTAX score, Electrocardiogram, Risk stratification

## Abstract

**Background:**

SYNTAX score is one of the risk assessment systems to predict cardiac events in acute coronary syndrome patients. Despite the large number of SYNTAX score benefits, invasive methods such as coronary angiography are necessary to perform the scoring. We hypothesized that ECG parameters could predict the SYNTAX score in unstable angina patients.

**Methods:**

During the retrospective cohort study, a total number of 876 patients were diagnosed with unstable angina. After applying the exclusion criteria, 600 patients were divided into tertiles based on the SYNTAX scores as low (0–22), intermediate (23–32), and high (≥ 33). The association between ECG parameters and SYNTAX score was investigated.

**Results:**

The study included 65% men and 35% women with a mean age of 62.4 ± 9.97 years. The delayed transition zone of QRS complex, ST-depression in inferior-lateral territories or/and in all three territories, and T-wave inversion in lateral territory were significant (*p* < 0.05) independent predictors of intermediate SYNTAX score. High SYNTAX score was predicted by the presence of prolonged P wave duration, ST-depression in lateral territory or/and anterior-lateral territories, ST-elevation in aVR–III leads or/and aVR–III–V1 leads. Among those, all three territories ST-depression (AUC: 0.611, sensitivity: 75%, specificity: 51%) and aVR + III ST-elevation (AUC: 0.672, sensitivity: 50.12%, specificity: 80.50%) were the most accurate parameters to predict intermediate and high SYNTAX scores, respectively.

**Conclusion:**

The present study demonstrates that accompanying the STE in the right side leads (aVR, III, V1) with ST-depression in other leads indicates the patients with high SYNTAX score; meanwhile, diffuse ST-depression without ST-elevation is a marker for intermediate SYNTAX score in unstable angina patients and can be applied for early risk stratification and intervention.

## Background

In the beginning, the SYNTAX score has been introduced as an anatomic tool that helps cardiologists and cardiac surgeons to figure out the complexity of coronary artery disease (CAD). Decision-making about choosing the proper modality for the revascularization between the percutaneous coronary intervention (PCI) and coronary artery bypass grafting (CABG) was facilitated by the SYNTAX score [1]. With the increase in CAD patients who underwent revascularization, the demand for risk stratification to predict the outcome was felt. Amongst the emergence of risk scoring systems, e.g. TIMI or GRACE score, SYNTAX score has established itself as an instrument to predict major adverse cardiac events (MACE) or cerebrovascular events [2–6]. However, a modified model of SYNTAX score comprising the clinical variables was provided later [5], but the need for angiography as an invasive procedure to calculate the scoring still existed. An established association between the SYNTAX score and non-invasive parameters leads to obtaining the SYNTAX score benefits without angiography. Previous investigations illustrated the association between non-invasive parameters such as laboratory variables and SYNTAX score [7–10], but none of them scrutinized the relationship of electrocardiogram (ECG) components and SYNTAX score. Patients with unstable angina (UA) should undergo early risk stratification to identify the need for an early invasive strategy to decrease the mortality rate and rehospitalization, and eventually lower medical expenditure.

To the best of our knowledge, no previous study has examined the association between ECG parameters and SYNTAX score in patients with UA. We hypothesized that ECG parameters could effectively predict the SYNTAX score. Therefore, we conducted the study to elucidate the utility of the SYNTAX score predictability by ECG parameters in patients with UA.

## Material and methods

### Study population

A retrospective cohort single-center study was conducted at Al-Zahra charity hospital, a university-affiliated tertiary medical center in Shiraz, for the period of 2 years from 2019 to 2021. The university ethics committee approved the study protocol; all the study procedures were conducted following the Declaration of Helsinki and all patients gave written informed consent before the study.

Inclusion criteria consisted of being diagnosed with UA and having informed consent. UA was diagnosed upon meeting at least one of the following eligibility criteria according to Braunwald:

1. Occurring at rest or minimal exertion and usually lasting > 20 min (if not interrupted with nitroglycerine administration)
2. Being new-onset (within 1 month) and severe and described as frank pain
3. Occurring with a crescendo pattern (more severe, prolonged, or frequent than before), in the absence of signs of ST-segment elevation myocardial infarction (STEMI) in left-sided, right-sided, and posterior ECG, and/or positive cardiac biomarkers.

The subjects were excluded in case of (1) Prior revascularization therapy (either PCI or CABG) (2) Valvular heart disease, (3) Paced rhythm or any kind of arrhythmia e.g. atrial fibrillation or Wolff-Parkinson-White Syndrome, (4) Any conductive delays such as sinus node disease, atrioventricular conductive block, bundle branch block, and (5) Those with low quality of ECG recording or missing data.

After patient selection, demographic data, history of smoking, risk factors for hypertension, dyslipidemia, diabetes mellitus, and stroke of patients were acquired from the medical records, and an initial ECG at the emergency room was recorded for subsequent analysis. Those with baseline blood pressure more than 140/90 mm Hg or under treatment with antihypertensive drugs were diagnosed with hypertension. Patients with diabetes mellitus were defined as pre-diagnosed, and/or receiving antidiabetic medications, or newly diagnosed if fasting plasma glucose was ≥ 126 mg/dL or blood glucose was ≥ 200 mg/dL at any time. Dyslipidemia was defined as a serum total cholesterol concentration of ≥ 220 mg/dL, low-density lipoprotein cholesterol ≥ 140 mg/dL, or the need for treatment with lipid-lowering agents. Positive history of stroke was defined as having at least one ischemic cerebrovascular event that persisted for ≥ 24 h and was diagnosed by a neurologist [11].

### Angiographic analyses

Coronary angiography was done via the femoral route by an experienced cardiologist who was blinded to the patients' name. SYNTAX score was calculated online by the most recently updated version (www.syntaxscore.com). Thereafter, the SYNTAX score was divided into tertiles as low (0–22), intermediate (23–32), and high (≥ 33), based on the SYNTAX trial [12].

### Electrocardiographic analyses

A prospective analysis of the ECG recorded at the first visit to the emergency room was performed to determine the normality of P wave, QRS complex, ST segment, and T wave. All ECGs included 8 s of data digitally recorded at a speed of 25 mm/s, amplitude of 10 mm/mv (cardiax system 4.25.5). The normal P wave is positive in leads I and II, commonly biphasic in lead V1, less than 3 small squares (0.12 s) in duration, and less than 2.5 small squares (0.25 mV) in amplitude.

Delays in ventricular depolarization (for example, bundle branch block) give rise to abnormally wide QRS complexes (≥ 0.12 s). The normal adult QRS axis is between − 30° and + 90°. If the QRS axis falls between − 30° and − 90°, the presence of left axis deviation is considered. QRS axis falling between + 90° and 180°, or beyond + 100° if the adult range is used, indicates the occurrence of right axis deviation. The lead with an equiphasic QRS complex is located over the transition zone; this normally lies between the leads V3 and V4, but if it lies before V3–V4 or after V3–V4, early or delayed transitional zone is assumed, respectively. Regarding the left and right ventricular hypertrophy (LVH and RVH), the most notable and widely used criterion is based on Sokolow and Lyon definition: the sum of S_V1_and R_V5_ or R_V6_ > 35 mm is a diagnostic criterion for LVH, and the sum of R_V1_ and S_V5,6_ > 10.5 mm is used as a diagnostic criterion for RVH [13–15].

As stated earlier, patients with the sign of STEMI in ECG were excluded; yet, ST elevation (STE) in right-side leads (aVR, V_1_, III) may appear as reciprocal changes and a result of ST-depression (STD) in precordial leads. To evaluate the STE clinically, generally accepted threshold limits exist. A threshold value of 0.25 mV and 0.2 mV should be considered for males < 40 years old and males ≥ 40 years old, respectively, and for adult females the value is 0.15 mV in V_2_ and V_3_ leads. In all the other standard leads, the upper limit of normal J-point elevation for both males and females is 0.1 mV. Moreover, for both males and females the J-point depression threshold values are − 0.1 mV in all leads. Values of STE or STD more than the defined threshold are considered clinically significant [16].

T wave inversion (TWI) was defined as "T wave showing ≥ 1 mm negative deflection from the isoelectric line" [17]. To consider STD and TWI in each of the anterior territory (V1–V4), inferior territory (II, III, and aVF), and lateral territory (I, aVL, V5, and V6), their presence should be in at least two contiguous leads.

### Statistical analyses

The statistical analysis was performed using the SPSS V.23.0 software package. Categorical data were presented by numbers and percentages [%]; mean ± standard deviation was used for continuous data. The statistical significance of the differences between groups of patients in continuous parameters was tested using the one‑way ANOVA test. Comparisons between binary parameters were made using the Chi-square test or the Fisher exact test. The relationship between intermediate and high SYNTAX scores with their potential predictors was analyzed by univariate logistic regression models and illustrated by odds ratios, their 95% confidence interval (CI), and corresponding statistical significance. Subsequently, multivariate models working with predictors statistically significant in univariate analysis were constructed. Receiver operating characteristic (ROC) curves were used to determine the accuracy, sensitivity, and specificity of significant parameters (the result of multivariant analysis) to predict the SYNTAX scores. The results of all analyses were considered significant if a P value less than 0.05 was obtained.

## Results

**Fig. 1:**
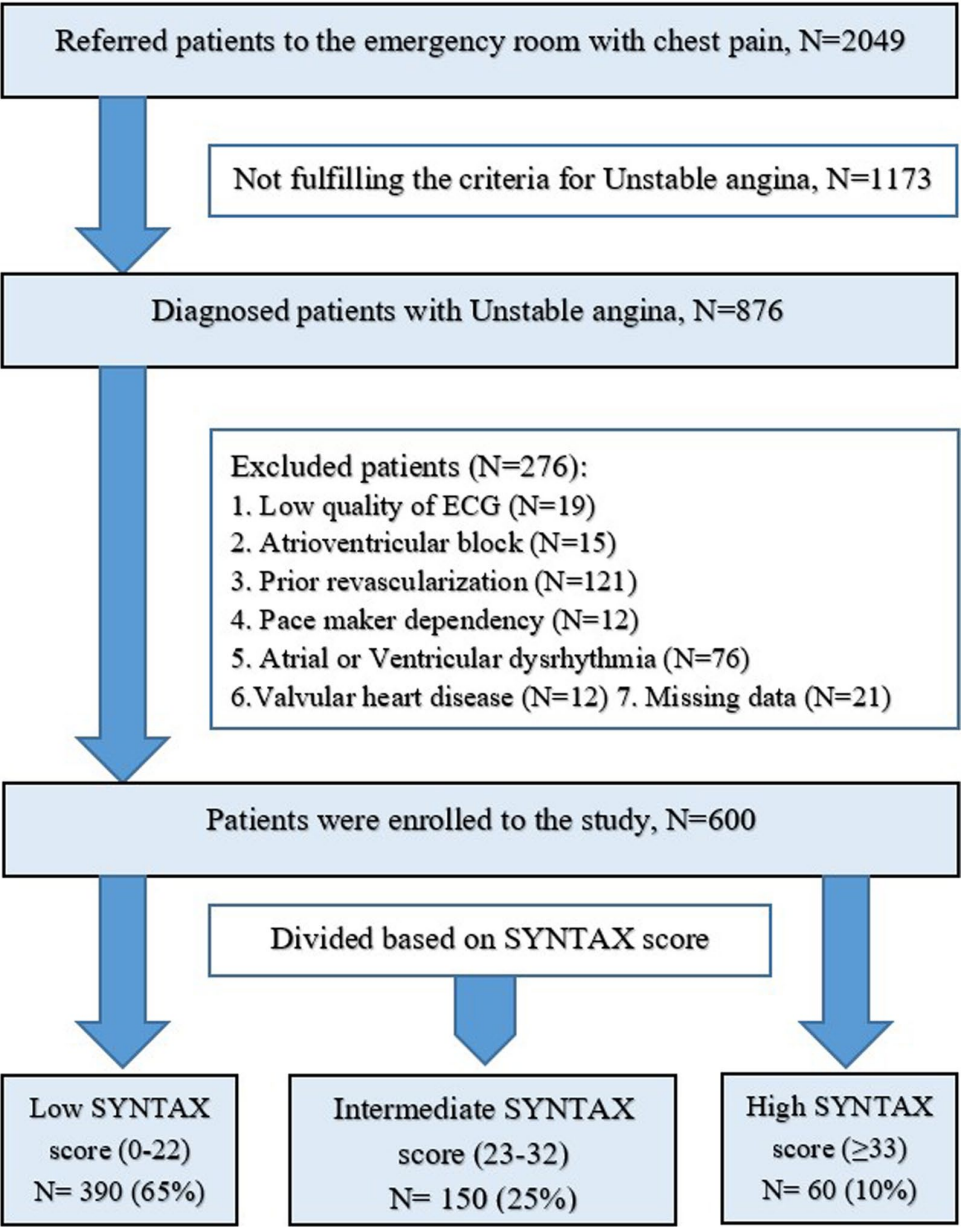
Flowchart of patient selection. A total number of 2049 patients were diagnosed with chest pain, and 876 of them had unstable angina. Data of 600 patients were analyzed after applying the exclusion criteria, and the patients were divided into tertiles based on SYNTAX score as low (0–22), intermediate (23–32), and high (≥ 33)

The flowchart of patient selection is presented in Fig. 1. During the 2-year study period, 2049 patients were referred to the emergency department with chest pain; among them, 876 consecutive patients were diagnosed with UA. Thus, they were enrolled in the study. Two hundred seventy-six patients were excluded after applying the exclusion criteria. As a result, the remaining 600 patients were divided into low, intermediate, and high SYNTAX scores as defined before, and data of them were analyzed. Demographic and clinical data of the patients are shown in Table 1. The study sample consisted of 390(65%) men and 210(35%) women with a mean age of 62.4 ± 9.97 years. Smoking was revealed in 117 (19%) of patients; meanwhile, opium was used in 63(10.5%) of them as a substance. Hypertension (61%), diabetes mellitus (36%), and dyslipidemia (36%) were the most significant comorbidities in the participants. The prevalence of cardiovascular risk factors in this population from Iran is similar to that of other large contemporary trials [18] and real-world registries [19] including other ethnicities. This is a relevant finding and potentially supports the generalizability of the results. Concerning demographic features, comorbidity, and vital status, no statically significant differences between the SYNTAX score tertile groups were shown. Significant differences (*p* value < 0.05) regarding ECG parameters in UA patients within SYNTAX groups are summarized in Table 1.

**Table 1:** Baseline characteristics of the patients based on the tertiles of the SYNTAX score

Characteristic	SYNTAX score				*p* value^a^
	All patients	Low (n = 390)	Intermediate (n = 150)	High (n = 60)	
Age, year	62.4 ± 9.97	61.19 ± 9.90	64.82 ± 9.77	64.20 ± 10.1	0.064
Male, n (%)	390 (65%)	267 (68.5%)	81 (20.8%)	42 (10.8%)	0.168
Female, n (%)	210 (35%)	123 (58.6%)	69 (32.9%)	18 (8.6%)	
Current smoker, n (%)	117 (19%)	75 (64.1%)	33 (28.2%)	9 (7.7%)	0.793
Substance user, n (%)	63 (10.5%)	48 (76.2%)	12 (19%)	3 (4.8%)	0.490
*Comorbidity, n (%)*					
Diabetes mellitus	216 (36%)	144 (66.7%)	51 (23.6%)	21 (9.7%)	0.931
Hypertension	369 (61%)	234 (63.4%)	99 (26.8%)	36 (9.8%)	0.752
Dyslipidemia	216 (36%)	141 (65.3%)	57 (26.4%)	18 (8.3%)	0.818
Stroke	30 (5%)	27 (90%)	0 (0%)	3 (10%)	0.162
*Vital status*					
SBP (mm Hg)	129.9 ± 27.2	129.3 ± 27.4	128.4 ± 27.8	134.8 ± 24.3	0.280
DBP (mm Hg)	80.01 ± 15.8	80.01 ± 16.0	79.69 ± 16.5	80.80 ± 12.6	0.901
HR (per minute)	90.7 ± 18.3	89.56 ± 17.0	92.33 ± 19.3	94.00 ± 22.9	0.099
RR (per minute)	17.36 ± 3.0	17.43 ± 2.9	17.25 ± 3.3	17.18 ± 2.6	0.748
*ECG parameters*					
*P wave duration, n (%)*					
Normal	569 (94.8%)	376 (65%)	141 (25%)	52 (10%)	**0.006**
Prolonged	31 (5.2%)	14 (45.2%)	9 (29%)	8 (25.8%)	
*QRS Complex, n (%)*					
Normal widening	570 (95%)	372 (65.3%)	144 (25.3%)	54 (9.5%)	0.614
LBBB	21 (3.5%)	12 (57.1%)	6 (28.6%)	3 (14.3%)	
RBBB	9 (1.5%)	6 (66.7%)	0 (0%)	3 (33.3%)	
Normal axis	585 (97.5%)	381 (65.1%)	147 (25.1%)	57 (9.7%)	0.810
Left axis deviation	12 (2%)	6 (50%)	3 (25%)	3 (25%)	
Right axis deviation	3 (0.5%)	3 (100%)	0 (0%)	0 (0%)	
Normal transition zone	486 (81%)	321 (66%)	84 (23.5%)	51 (10.5%)	0.331
Early transition zone	66 (11%)	42 (63.6%)	15 (22.7%)	9 (13.6%)	
Delayed transition zone	48 (8%)	27 (56.3%)	21 (43.8%)	0 (0%)	
Without hypertrophy	567 (94.5%)	366 (64.6%)	141 (24.9%)	60 (10.6%)	0.692
LVH	21 (4%)	12 (57.1%)	9 (42.9%)	0 (0%)	
RVH	6 (1.5%)	6 (100%)	0 (%)	0 (0%)	
LVH + RVH	*6* (1.5%)	6 (100%)	0 (0%)	0 (0%)	
*ST-Segment, n (%)*					
Without depression	345 (57.5%)	252 (73%)	75 (21.7%)	18 (5.2%)	**< 0.001**
ST depression	255 (42.5%)	138 (54.1%)	75 (29.4%)	42 (16.5%)	
Anterior leads	6 (1%)	3 (50%)	3 (50%)	0 (0%)	0.312
Inferior leads	15 (2.5%)	12 (80%)	0 (0%)	3 (20%)	0.502
Lateral leads	48 (8%)	21 (43.8%)	15 (31.3%)	12 (25%)	**< 0.001**
Anterior + Inferior leads	3 (0.5%)	3 (100%)	0 (0%)	0 (0%)	0.444
Inferior + Lateral leads	114 (19%)	54 (47.4%)	48 (42.1%)	12 (10.5%)	**< 0.001**
Anterior + Lateral leads	33 (5.5%)	18 (54.5%)	6 (18.2%)	9 (27.3%)	**0.003**
Anterior + Lateral + Inferior leads	36 (6%)	27 (75%)	3 (8.3%)	6 (16.7%)	**0.038**
Without elevation	414 (69%)	291 (70.2%)	99 (23.9%)	24 (5.7%)	**< 0.001**
ST Elevation	186 (31%)	99 (53.2%)	51 (27.4%)	36 (19.3%)	
AVR lead	75 (12.5%)	51 (68%)	15 (20%)	9 (12%)	0.517
V_1_ lead	3 (0.5%)	0 (0%)	0 (0%)	3 (100%)	**< 0.001**
III lead	6 (1%)	3 (50%)	3 (50%)	0 (0%)	0.312
AVR + V_1_ leads	66 (11%)	33 (50%)	24 (36.4%)	9 (13.6%)	**0.025**
AVR + III leads	18 (3%)	3 (16.7%)	3 (16.7%)	12 (66.7%)	**< 0.001**
V_1_ + III leads	9 (1.5%)	6 (67%)	3 (33%)	0 (0%)	0.557
AVR + V_1_ + III leads	9 (1.5%)	3 (33.3%)	3 (33.3%)	3 (33.3%)	**0.036**
*T wave, n (%)*					
Normal T wave	495 (82.5%)	315 (63.6%)	132 (26.7%)	48 (9.7%)	0.723
Inverted	105 (17.5%)	75 (71.4%)	18 (17.1%)	12 (11.4%)	
Lateral leads	48 (45.7%)	39 (81.3%)	6 (12.5%)	3 (6.3%)	**0.047**
Anterior leads	18 (17.1%)	9 (50%)	6 (33.3%)	3 (16.7%)	0.396
Inferior leads	39 (37.1%)	27 (69.2%)	6 (15.4%)	6 (15.4%)	0.238

All statistically significant p values (*p* < 0.05) are in bold

*SBP* Systolic blood pressure, *DBP* diastolic blood pressure, *HR* heart rate, *RR* respiratory rate, *RBBB* right bundle branch block, *LBBB* left bundle branch block, *TZ* transitional zone, *LVH* left ventricular hypertrophy, *RVH* right ventricular hypertrophy

^a^ Statistical significance of (1) binary parameters in the chi-square test or Fisher exact test as appropriate (2) continuous parameters in one-way ANOVA test

**Fig. 2:**
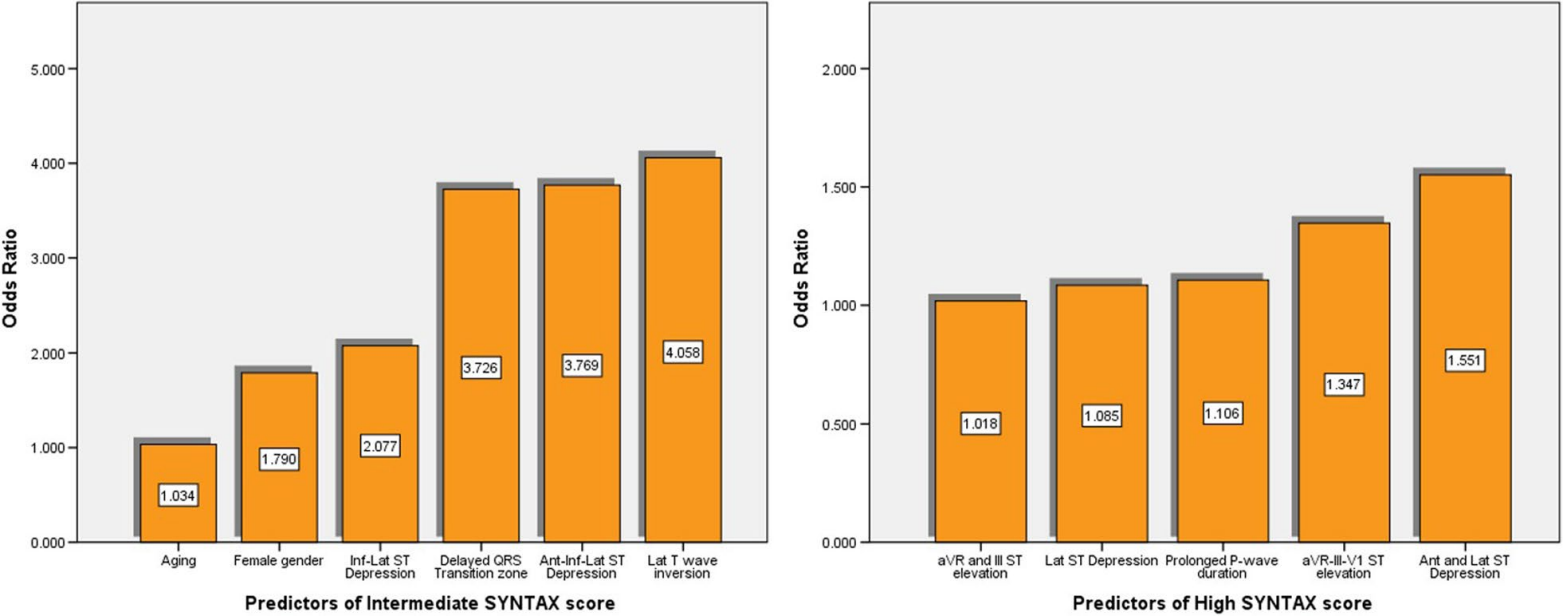
Independents predictors of SYNTAX scores

The predictive accuracy of the variables derived from demographic characteristics, comorbidity, vital status, and ECGs parameters was investigated initially using univariate and then multivariate analysis concerning intermediate and high SYNTAX scores (Table 2). Aging [OR 1.034 (increase of risk per 1 year of age), 95% CI 1.012–1.055, *p* = 0.002], female gender [OR 1.790 (increase of risk compared to male gender, 95%CI 1.186–2.702, *p* = 0.006], delayed transition zone of QRS complex [OR 3.726 (increase of risk compare to normal or early QRS transition zone), 95% CI 1.903–7.295, *p* = 0.001], STD in inferior-lateral territories [OR 2.077, 95% CI 1.272–3.391, *p* = 0.003], STD in all three territories [OR 3.769, 95%CI 1.054–13.47, *p* = 0.041], and TWI in lateral territory [OR 4.058, 95% CI 1.560–10.55, *p* = 0.004] were the independent predictors of intermediate SYNTAX score in multivariate analysis (Fig. 2).

**Table 2:** Univariate and multivariate analysis of significant predictors of intermediate and high SYNTAX score

	Univariate analysis		Multivariate analysis	
	OR (95% CI)^b^	*P*^a^	OR (95% CI)^b^	*P*^a^
*Significant predictors of Intermediate SYNTAX score*				
Age	1.034 (1.014; 1.054)	**0.001**	1.034 (1.012; 1.055)	**0.002**
Sex	1.867 (1.297; 2.724)	**0.001**	1.790 (1.186; 2.702)	**0.006**
Delayed QRS TZ	2.538 (1.382; 4.660)	**0.019**	3.726 (1.903; 7.295)	**0.001**
Inf-Lat ST Depression	2.738 (1.779; 4.214)	**< 0.001**	2.077 (1.272; 3.391)	**0.003**
Ant-Inf-Lat ST Depression	3.878 (1.172; 9.833)	**0.026**	3.769 (1.054; 13.47)	**0.041**
aVR and V_1_ ST-elevation	1.850 (1.078; 3.175)	**0.025**	1.471 (0.767; 2.823)	0.246
Lat T wave inversion	2.471 (1.029; 5.934)	**0.043**	4.058 (1.560; 10.55)	**0.004**
*Significant predictors of High SYNTAX score*				
Prolonged P-wave duration	1.234 (1.079; 1.409)	**0.004**	1.106 (1.009; 1.210)	**0.045**
Prolonged QRS duration	1.593 (1.162; 2.019)	**0.030**	2.851 (0.085; 8.527)	0.891
Lat ST Depression	1.041 (1.015; 1.067)	**0.001**	1.085 (1.023; 1.151)	**0.001**
Ant and Lat ST Depression	2.636 (1.551; 4.432)	**0.001**	1.551 (1.373; 1.776)	**0.029**
aVR and III ST elevation	1.063 (1.023; 1.105)	**0.032**	1.018 (1.005; 1.031)	**0.001**
aVR–III–V1 ST elevation	1.092 (1.006; 1.185)	**< 0.001**	1.347 (1.043; 1.671)	**0.043**

All statistically significant *p* values (*p* < 0.05) are in bold

*TZ* Transition zone, *Ant* Anterior, *Inf* Inferior, *Lat* Lateral

^a^Statistical significance of odds ratio

^b^Odds ratio calculated by uni and multivariate logistic regression of intermediate and high SYNTAX score and its 95% confidence interval

At the same time, High SYNTAX score was predicted by presence of prolonged PWD [OR OR 1.106, 95% CI 1.009–1.210, *p* = 0.045], STD in lateral territory [OR 1.085, 95%CI 1.023–1.151, *p* = 0.001] or/and anterior-lateral territories [OR 1.551, 95% CI 1.373–1.776, *p* = 0.029], STE in aVR–III leads [OR 1.018, 95% CI 1.005–1.031, *p* = 0.001] or/and aVR–III–V1 leads [OR 1.347, 95% CI 1.043–1.671, *p* = 0.043] in multivariate regression model (Fig. 2).

Significant variables of multivariate regression model were analyzed by ROC curves to determine their accuracy, sensitivity, and specificity (Table 3). Area under the ROC Curve(AUC) [(95% CI), *p* value, sensitivity, and specificity] of parameters to predict Intermediate SYNTAX score were 0.507 [(0.466–0.547), 0.643, 90%, and 11.33%] for delayed QRS transition zone, 0.596 [(0.545–0.646), 0.017, 71.93%, and 50.46%] for inferior-lateral STD, 0.611 [(0.560–0.660), 0.007, 75.44%, and 51.70%] for all three territories STD, 0.532 [(0.480–0.583), 0.146, 76%, and 19.33%] for lateral T-wave inversion.

**Table 3:** Receiver operating characteristic (ROC) curve analysis for SYNTAX score prediction

	AUC (95% CI)	*p* value	Sensitivity (%)	Specificity (%)
*Intermediate SYNTAX score*				
Delayed QRS transition zone	0.507 (0.466–0.547)	0.643	90.00	11.33
Inf and Lat ST depression	0.596 (0.545–0.646)	**0.017**	71.93	50.46
Ant, Inf, and Lat ST depression	0.611 (0.560–0.660)	**0.007**	75.44	51.70
Lat T-wave inversion	0.532 (0.480–0.583)	0.146	76.00	19.33
*High SYNTAX score*				
Prolonged P-wave duration	0.515 (0.475–0.556)	0.244	33.33	55.74
Lat ST depression	0.588 (0.537–0.638)	**0.019**	89.47	33.33
Ant and Lat ST depression	0.592 (0.541–0.642)	**0.026**	87.72	36.93
aVR and III ST elevation	0.672 (0.622–0.719)	**< 0.001**	50.12	80.50
aVR, III, and V_1_ ST elevation	0.519 (0.479–0.560)	0.175	15.00	98.89

*AUC* area under the curve, *CI* confidence interval, *Ant* anterior, *Inf* inferior, *Lat* lateral

^*^All statistically significant *p* values (*p* < 0.05) are in bold

**Fig. 3:**
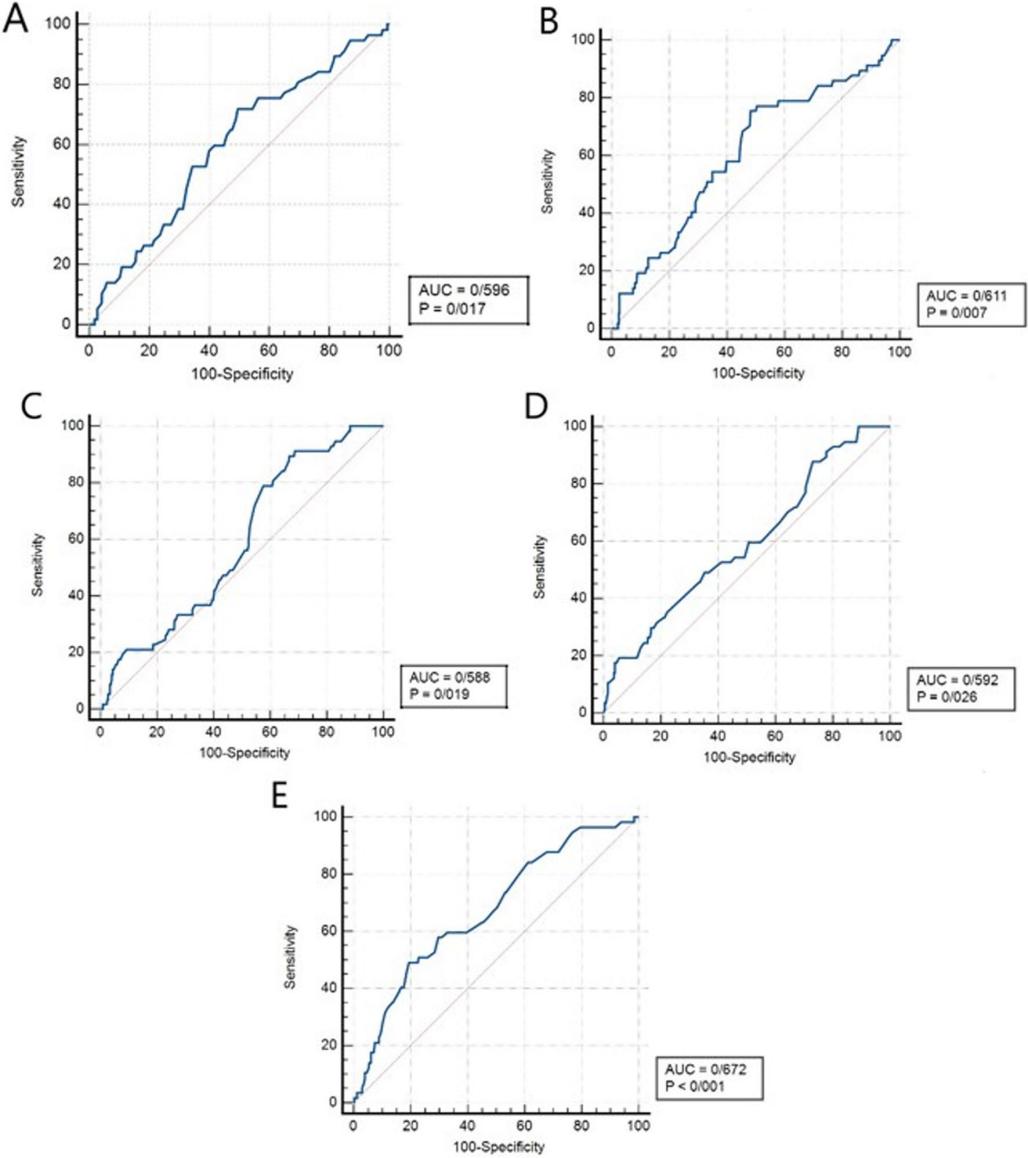
The receiver operating characteristic curve for the significant predictors of Intermediate (**a** Inferior-Lateral ST depression; **b** All three territories ST depression) and High SYNTAX score (**c** Lateral ST depression; **d** Anterior-Lateral ST depression; **e** aVR + III ST elevation)

Also, the amounts of 0.515 [(0.475–0.556), 0.244, 33.33%, and 55.74%] for prolonged P-wave duration, 0.588 [(0.537–0.638), 0.019, 89.47%, and 33.33%] for lateral STD, 0.592 [(0.541–0.642), 0.026, 87.72%, and 36.93%] for anterior-lateral territories STD, 0.672 [(0.622–0.719), *p* < 0.001, 50.12%, and 80.50%] for aVR + III STE, 0.519 [(0.479–0.560), 0.175, 15%, and 98.89%] for aVR + III + V_1_ STE were acquired by ROC analysis of High SYNTAX score (Fig. 3). It seems that all three territories STD for intermediate SYNTAX score and aVR + III STE for high SYNTAX score were the most accurate parameters for prediction.

## Discussion

We surveyed the predictive role of ECG parameters for the early risk stratification of the intermediate and high SYNTAX scores in patients with UA. The main discovery of our study was that accompanying the STE in the right side leads (aVR, III, V1) with STD in other leads indicates the patients with high SYNTAX score; meanwhile, diffuse depression of ST-segment without STE is a marker for intermediate SYNTAX score.

Early risk stratification to perform rapid intervention is necessary. Risk stratification in acute coronary syndrome (ACS) has been studied to compare between STEMI, Non-STEMI, and UA subset groups [20, 21] or just in STEMI and Non-STEMI [22–24]; nevertheless, few studies have targeted early risk stratification among UA patients.

Despite the established role of SYNTAX score as an anatomical based tool used for objectively determining the complexity of CAD and guiding decision-making between CABG and PCI [12, 17], literature has suggested that angiographic variables and SYNTAX score should be more widely used in risk stratification or prediction of mortality and MACE in patients with ACS [2–6]. However, despite having a large number of benefits, the need to perform an invasive method such as coronary angiography is considered a drawback of the SYNTAX score.

Utility of various tools such as layer-specific strain in echocardiography [7], C-reactive protein (CRP) level [8], high-sensitive troponin T, the neutrophil–lymphocyte ratio [10], or thickness of the carotid layer [9] to predict the SYNTAX score has been already demonstrated, but ECG as an easily accessible, relatively inexpensive, simple to perform, objective and reproducible tool that gives instant results could be more useful for prediction.

### Interpretation of ECG parameters

#### P wave

Two mechanisms have been hypothesized to explain the prolongation of PWD in severe CAD. First, CAD may result in atrial ischemia. Second, left ventricular ischemia causes systolic and diastolic dysfunction that results in increased left ventricular end-diastolic pressure. Eventually, the consequent increased left atrium pressure and/or volume loading appears in the prolongation of atrial depolarization time [25]. Investigations [22, 26] indicated the usefulness of P wave peak time and PWD to predict the ischemic events and severity of CAD. In accordance with previous studies, a predictive role of prolonged PWD for the high SYNTAX score was found in our investigation.

#### QRS complex

Expanded atherosclerosis may involve the ventricular myocytes and Purkinje system, leading to delay in conduction velocity and prolonged QRS [17, 22, 27, 28]. New parameters related to QRS complex including fragmented QRS [27, 29], R wave peak time [17], and ventricular repolarization indicators such as corrected QT dispersion [30], or Tp-e interval, Tp-e/QT, Tp-e/QTc ratios [31] have been introduced as predictors of high SYNTAX score. In contrast, our efforts to corroborate a significant association between QRS complex parameters with intermediate and high SYNTAX scores failed, except for the delayed QRS transition zone; to the best of our knowledge, this is the first study to show that the delayed QRS transition zone is an independent predictor of intermediate SYNTAX score.

#### ST-segment

ST-segment changes, including STD and STE in aVR lead, have been shown as predictors of high SYNTAX score or three-vessel disease in patients with ACS [17, 22, 28, 32]. Consistent with previous studies, our results show that (1) STD and STE are related to SYNTAX score; nevertheless, the differentiating point between intermediate and high SYNTAX scores is based on the presence of STE changes in reciprocal leads. (2) The number of territories or leads demonstrating either STD or STE is associated with the power of prediction.

Three-vessel disease and left circumflex coronary (LCX) artery involvement are more commonly involved in non-STEMI/UA rather than STEMI patients [33], and ischemic changes due to culprit lesion in LCX are illustrated in lateral leads of ECG [34]. Thus, lateral leads have a crucial role in CAD in UA patients. Persistent with this result, our research suggests that on condition that lateral leads are involved, STD would be an independent predictor of both intermediate and high SYNTAX in one, two, and/or three involved territories.

Previous studies have suggested that STE in aVR lead, with or without STE in V_1_ lead, is a predictor of high SYNTAX score [35] or left main/3VD [32, 35, 36]. In our study, we included lead III as well as aVR and V1 as the right-side leads that indicate reciprocal changes of sub-endocardial ischemia. Opposite to STD, predictive role of STE was concluded only for high SYNTAX score and not in intermediate on condition that two or three reciprocal leads (one of which must be aVR as the most significant reciprocal lead) are involved. Ischemic heart disease is one of the clinical presentations of atherosclerosis [37], and the level of ischemic changes are related to atherosclerosis burden. Furthermore, a significant association between atherosclerosis burden and SYNTAX score, a marker of CAD complexity, has been previously illustrated [38]. Therefore, the SYNTAX score depends on the severity of ischemic changes. Following the stated conclusion, our result showed that the strength of association between ST-segment changes and each group of SYNTAX score was obtained from the number of involved territories and leads. If STE is observed in three reciprocal leads compared to two and/or if STD is demonstrated in three territories compared to two, there will be a higher probability of intermediate and high SYNTAX score occurrence, respectively.

#### T wave

Regarding the predictive value of T wave in CAD, most of the articles are dedicated to the predictive role of positive T wave [35, 36], and few investigations are done to elucidate the correlation of negative T wave with SYNTAX score or severity of CAD. A previous study has reported that TWI in aVL lead is correlated with the severity of stenosis in the left anterior descending artery [39]. While Rencüzoğulları et al. [17] highlighted that TWI was not associated with SYNTAX score, but the presence of TWI in lateral leads had a significant relationship with intermediate SYNTAX in our work.

### Limitations

Because of the retrospective design of this study, our investigation did not include prognostic information. Also, conducting the study on a single-center may influence its external validity. In addition, exclusion of systolic and diastolic dysfunction, which affects the atrial and ventricular filling pressure and eventually sub-endocardium, was not done. Moreover, determination of SYNTAX score was done visually while performing coronary angiography. The clinical application of our findings is dedicated to those who have not undergone PCI or CABG, so this study's results cannot be applied to the overall CAD population. It should also be noted that other conditions such as medications (e.g. antiarrhythmic drugs) or electrolyte imbalance can be associated with ST-segment and T-wave changes which were not considered in the present study.

## Conclusion

Recognizing the patients with severe CAD or intermediate and high SYNTAX scores using non-invasive tools is extremely helpful, especially for centers without advanced facilities. ECG can be used for this purpose. The present study demonstrates that accompanying the STE in the right side leads (aVR, III, V1) with STD in other leads indicates the patients with high SYNTAX score; meanwhile, diffuse depression of ST-segment without STE is a marker for intermediate SYNTAX score. These predictors aid us in early risk stratification and intervention.

## Data Availability

The datasets used and/or analyzed during the current study are available from the corresponding author on reasonable request.

## Abbreviations

CAD: Coronary artery disease
PCI: Percutaneous coronary intervention
CABG: Coronary artery bypass grafting
MACE: Major adverse cardiac events
ECG: Electrocardiogram
UA: Unstable angina
STEMI: ST-segment elevation myocardial infarction
LVH: Left ventricular hypertrophy
RVH: Right ventricular hypertrophy
STE: ST elevation
STD: ST depression
TWI: T wave inversion
CI: Confidence interval
ROC: Receiver operating characteristic
PWD: P wave duration
LBBB: Left bundle branch block
ACS: Acute coronary syndrome
LCX: Left circumflex coronary

## References

[1] Sianos G, Morel MA, Kappetein AP, Morice MC, Colombo A, Dawkins K, van den Brand M, Van Dyck N, Russell ME, Mohr FW, Serruys PW (2005). The SYNTAX Score: an angiographic tool grading the complexity of coronary artery disease. EuroInterv J EuroPCR Collab Work Group Interv Cardiol Eur Soc Cardiol.

[2] Safarian H, Alidoosti M, Shafiee A, Salarifar M, Poorhosseini H, Nematipour E (2014). The SYNTAX score can predict major adverse cardiac events following percutaneous coronary intervention. Heart Views Off J Gulf Heart Assoc.

[3] Minamisawa M, Miura T, Motoki H, Kobayashi H, Kobayashi M, Nakajima H, Kimura H, Akanuma H, Mawatari E, Sato T, Hotta S, Kamiyoshi Y, Maruyama T, Watanabe N, Eisawa T, Aso S, Uchikawa S, Senda K, Morita T, Hashizume N, Abe N, Ebisawa S, Izawa A, Miyashita Y, Koyama J, Ikeda U (2017). Prediction of 1-year clinical outcomes using the SYNTAX score in patients with prior heart failure undergoing percutaneous coronary intervention: sub-analysis of the SHINANO registry. Heart Vessels.

[4] Maciejewski P, Lewandowski P, Wąsek W, Budaj A (2013). Assessment of the prognostic value of coronary angiography in patients with non-ST segment elevation myocardial infarction. Kardiol Polska.

[5] Garg S, Sarno G, Garcia-Garcia HM, Girasis C, Wykrzykowska J, Dawkins KD, Serruys PW (2010). A new tool for the risk stratification of patients with complex coronary artery disease: the Clinical SYNTAX Score. Circ Cardiovasc Interv.

[6] Farooq V, Head SJ, Kappetein AP, Serruys PW (2014). Widening clinical applications of the SYNTAX Score. Heart (Br Cardiac Soc).

[7] Zhang L, Wu WC, Ma H, Wang H (2016). Usefulness of layer-specific strain for identifying complex CAD and predicting the severity of coronary lesions in patients with non-ST-segment elevation acute coronary syndrome: compared with Syntax score. Int J Cardiol.

[8] Karadeniz M, Duran M, Akyel A, Yarlıoğlueş M, Öcek AH, Çelik İE, Kılıç A, Yalcin AA, Ergün G, Murat SN (2015). High sensitive CRP level is associated with intermediate and high syntax score in patients with acute coronary syndrome. Int Heart J.

[9] Ikeda N, Kogame N, Iijima R, Nakamura M, Sugi K (2012). Carotid artery intima-media thickness and plaque score can predict the SYNTAX score. Eur Heart J.

[10] Altun B, Turkon H, Tasolar H, Beggı H, Altun M, Temız A, Gazı E, Barutcu A, Bekler A, Colkesen Y (2014). The relationship between high-sensitive troponin T, neutrophil lymphocyte ratio and SYNTAX Score. Scand J Clin Lab Investig.

[11] Sacco RL, Kasner SE, Broderick JP, Caplan LR, Connors JJ, Culebras A, Elkind MS, George MG, Hamdan AD, Higashida RT, Hoh BL, Janis LS, Kase CS, Kleindorfer DO, Lee JM, Moseley ME, Peterson ED, Turan TN, Valderrama AL, Vinters HV (2013). An updated definition of stroke for the 21st century: a statement for healthcare professionals from the American Heart Association/American Stroke Association. Stroke.

[12] Head SJ, Davierwala PM, Serruys PW, Redwood SR, Colombo A, Mack MJ, Morice MC, Holmes DR, Feldman TE, Ståhle E, Underwood P, Dawkins KD, Kappetein AP, Mohr FW (2014). Coronary artery bypass grafting vs. percutaneous coronary intervention for patients with three-vessel disease: final five-year follow-up of the SYNTAX trial. Eur Heart J.

[13] Meek S, Morris F (2002). Introduction. II–basic terminology. BMJ (Clin Res ed).

[14] Kashou AH, Basit H, Chhabra L (2021). Electrical right and left axis deviation.

[15] Hancock EW, Deal BJ, Mirvis DM, Okin P, Kligfield P, Gettes LS, Bailey JJ, Childers R, Gorgels A, Josephson M, Kors JA, Macfarlane P, Mason JW, Pahlm O, Rautaharju PM, Surawicz B, van Herpen G, Wagner GS, Wellens H (2009). AHA/ACCF/HRS recommendations for the standardization and interpretation of the electrocardiogram: part V: electrocardiogram changes associated with cardiac chamber hypertrophy: a scientific statement from the American Heart Association Electrocardiography and Arrhythmias Committee, Council on Clinical Cardiology; the American College of Cardiology Foundation; and the Heart Rhythm Society. Endorsed by the International Society for Computerized Electrocardiology. J Am Coll Cardiol.

[16] Kashou AH, Basit H, Malik A (2021). ST segment.

[17] Rencüzoğulları İ, Çağdaş M, Karakoyun S, Karabağ Y, Yesin M, Artaç İ, İliş D, Selçuk M, Öterkuş M, Tanboğa H (2018). The association between electrocardiographic R wave peak time and coronary artery disease severity in patients with non-ST segment elevation myocardial infarction and unstable angina pectoris. J Electrocardiol.

[18] Valgimigli M, Gragnano F, Branca M, Franzone A, Baber U, Jang Y, Kimura T, Hahn JY, Zhao Q, Windecker S, Gibson CM, Kim BK, Watanabe H, Song YB, Zhu Y, Vranckx P, Mehta S, Hong SJ, Ando K, Gwon HC, Serruys PW, Dangas GD, McFadden EP, Angiolillo DJ, Heg D, Jüni P, Mehran R (2021). P2Y12 inhibitor monotherapy or dual antiplatelet therapy after coronary revascularisation: individual patient level meta-analysis of randomised controlled trials. BMJ (Clin Res ed).

[19] Cesaro A, Gragnano F, Calabrò P, Moscarella E, Santelli F, Fimiani F, Patti G, Cavallari I, Antonucci E, Cirillo P, Pignatelli P, Palareti G, Pelliccia F, Bossone E, Pengo V, Gresele P, Marcucci R, Schiavo A, Vergara A, Pastori D, Menichelli D, Grossi G, Di Serafino L, Taglialatela V, del Pinto M, Gugliemini G (2021). Prevalence and clinical implications of eligibility criteria for prolonged dual antithrombotic therapy in patients with PEGASUS and COMPASS phenotypes: Insights from the START-ANTIPLATELET registry. Int J Cardiol.

[20] Isilak Z, Kardesoglu E, Aparci M, Uz O, Yalcin M, Yiginer O, Cingozbay BY, Uzun M (2012). Comparison of clinical risk assessment systems in predicting three-vessel coronary artery disease and angiographic culprit lesion in patients with non-ST segment elevated myocardial infarction/unstable angina pectoris. Kardiol Polska.

[21] Ge J, Li J, Yu H, Hou B (2018). Hypertension is an independent predictor of multivessel coronary artery disease in young adults with acute coronary syndrome. Int J Hypertens.

[22] Burak C, Yesin M, Tanık VO, Çağdaş M, Rencüzoğulları İ, Karabağ Y, Hamideyin Ş, İliş D, Çınar T, Altıntaş B, Baysal E (2019). Prolonged P wave peak time is associated with the severity of coronary artery disease in patients with non-ST segment elevation myocardial infarction. J Electrocardiol.

[23] Carvalho JF, Belo A, Congo K, Neves D, Santos AR, Piçarra B, Damásio AF, Aguiar J (2018). Left main and/or three-vessel disease in patients with non-ST-segment elevation myocardial infarction and low-risk GRACE score: prevalence, clinical outcomes and predictors. Rev Port Cardiol Orgao Oficial Soc Port Cardiol Port J Cardiol Off J Port Soc Cardiol.

[24] Kosuge M, Ebina T, Hibi K, Morita S, Endo M, Maejima N, Iwahashi N, Okada K, Ishikawa T, Umemura S, Kimura K (2011). An early and simple predictor of severe left main and/or three-vessel disease in patients with non-ST-segment elevation acute coronary syndrome. Am J Cardiol.

[25] Alexander B, MacHaalany J, Lam B, van Rooy H, Haseeb S, Kuchtaruk A, Glover B, Bayés de Luna A, Baranchuk A (2017). Comparison of the extent of coronary artery disease in patients with versus without interatrial block and implications for new-onset atrial fibrillation. Am J Cardiol.

[26] Öz A, Cinar T, Kızılto Güler C, Efe SÇ, Emre U, Karabağ T, Ayça B (2020). Novel electrocardiography parameter for paroxysmal atrial fibrillation in acute ischaemic stroke patients: P wave peak time. Postgrad Med J.

[27] Bekler A, Barutçu A, Tenekecioglu E, Altun B, Gazi E, Temiz A, Kırılmaz B, Ozkan MT, Yener AU (2015). The relationship between fragmented QRS complexes and SYNTAX and Gensini scores in patients with acute coronary syndrome. Kardiol Polska.

[28] Kosuge M, Ebina T, Hibi K, Morita S, Komura N, Hashiba K, Kiyokuni M, Nakayama N, Umemura S, Kimura K (2009). Early, accurate, non-invasive predictors of left main or 3-vessel disease in patients with non-ST-segment elevation acute coronary syndrome. Circ J Off J Jpn Circ Soc.

[29] Yesin M, Çağdaş M, Kalçık M, Rencüzoğulları İ, Karabağ Y, Gürsoy MO, Karakoyun S (2018). The relationship between fragmented QRS complexes and syntax II scores in patients with ST-segment elevation myocardial infarction. J Electrocardiol.

[30] Helmy H, Abdel-Galeel A, Taha Kishk Y, Mohammed Sleem K (2017). Correlation of corrected QT dispersion with the severity of coronary artery disease detected by SYNTAX score in non-diabetic patients with STEMI. Egypt Heart J EHJ Off Bull Egypt Soc Cardiol.

[31] Kahraman S, Doğan A, Demirci G, Guler A, Kalkan A, Uzun F, Kurtoglu N, Erturk M, Kalkan M (2021). The association between Tp-e interval, Tp-e/QT, and Tp-e/QTc ratios and coronary artery disease spectrum and syntax score. Int J Cardiovasc Sci.

[32] Misumida N, Kobayashi A, Fox JT, Hanon S, Schweitzer P, Kanei Y (2016). Predictive value of ST-segment elevation in lead aVR for left main and/or three-vessel disease in non-ST-segment elevation myocardial infarction. Ann Noninvasive Electrocardiol Off J Int Soc Holter Noninvasive Electrocardiol.

[33] Deora S, Kumar T, Ramalingam R, Nanjappa Manjunath C (2016). Demographic and angiographic profile in premature cases of acute coronary syndrome: analysis of 820 young patients from South India. Cardiovasc Diagn Ther.

[34] Huey BL, Beller GA, Kaiser DL, Gibson RS (1988). A comprehensive analysis of myocardial infarction due to left circumflex artery occlusion: comparison with infarction due to right coronary artery and left anterior descending artery occlusion. J Am Coll Cardiol.

[35] İçen YK, Koç M (2017). ST segment change and T wave amplitude ratio in lead aVR associated with coronary artery disease severity in patients with non-ST elevation myocardial infarction: a retrospective study. Medicine.

[36] Separham A, Sohrabi B, Tajlil A, Pourafkari L, Sadeghi R, Ghaffari S, Nader ND (2018). Prognostic value of positive T wave in lead aVR in patients with non-ST segment myocardial infarction. Ann Noninvasive Electrocardiol Off J Int Soc Holter Noninvasive Electrocardiol.

[37] Roquer J, Ois A, Rodríguez-Campello A, Gomis M, Munteis E, Jiménez-Conde J, Cuadrado-Godia E, Martínez-Rodríguez JE (2007). Atherosclerotic burden and early mortality in acute ischemic stroke. Arch Neurol.

[38] Korkmaz L, Adar A, Korkmaz AA, Erkan H, Agac MT, Acar Z, Kurt IH, Akyuz AR, Celik S (2012). Atherosclerosis burden and coronary artery lesion complexity in acute coronary syndrome patients. Cardiol J.

[39] Behnemoon M, Sibi H (2020). T-wave inversion in the aVL lead is associated with severity of stenosis in the LAD artery. J Stud Med Sci.

